# Clinical efficacy of implantation of toric intraocular lenses with different incision positions: a comparative study of steep-axis incision and non-steep-axis incision

**DOI:** 10.1186/s12886-017-0528-x

**Published:** 2017-07-27

**Authors:** Wenwen He, Xiangjia Zhu, Yu Du, Jin Yang, Yi Lu

**Affiliations:** 1 Department of Ophthalmology, Eye and Ear, Nose, and Throat Hospital of Fudan University, 83 Fenyang Road, Shanghai, 200031 People’s Republic of China; 2 Key Laboratory of Myopia, Ministry of Health, Shanghai Medical College of Fudan University, Shanghai, 200031 People’s Republic of China; 3 Shanghai Key Laboratory of Visual Impairment and Restoration, Shanghai, 200031 People’s Republic of China

**Keywords:** Cataract surgery, Astigmatism, Incision, Toric intraocular lens, Visual function

## Abstract

**Background:**

To compare the clinical outcomes after implantation of AcrySof Toric IOLs with different clear corneal incisions.

**Methods:**

Sixty cataract patients with regular corneal astigmatism who underwent phacoemulsification combined with implantation of an AcrySof Toric IOL were enrolled. They were divided into two groups according to the position of the clear corneal incision: steep-axis and non-steep-axis. Main outcome measurements included visual acuity, residual astigmatism and changes of corneal astigmatism 3 months postoperatively. Deviation of IOL axis according to the re-calculation using the actual surgically induced astigmatism (SIA) and visual function 3 months after surgery were also evaluated.

**Results:**

The corneal astigmatism decreased significantly in steep-axis group 3 months postoperatively (*P* < 0.05). Besides, more patients in non-steep-axis group were with irregular corneal astigmatism after the surgery (43.33% vs 10%, *P* = 0.004). The deviation of IOL axis according to the re-calculation using the actual SIA was significantly larger in non-steep-axis group than that of steep-axis group (*P* < 0.001). Moreover, the postoperative visual function was better in eyes of steep-axis group in various measurements, including point spread function, modulation transfer function and high-order aberrations.

**Conclusion:**

Steep-axis clear corneal incision could reduce the cylinder power of toric IOL and lower the chance of postoperative irregular astigmatism, which may consequently improve the postoperative visual quality.

## Background

In virtue of improvements in surgical technology, the purpose of cataract surgery has advanced to the achievement of perfect visual quality postoperatively. However, 20%–30% of cataract patients, with corneal astigmatism of more than 1.25 diopters (D) before surgery [1, 2], cannot be perfectly treated with a normal, monofocal intraocular lens (IOL). Thus, these patients are usually implanted with a toric IOL.

Toric IOLs were first made and put into clinical practice by Shimizu et al. in 1994 [3]. The AcrySof Toric IOL (Alcon Laboratories, Inc., Fort Worth, Texas, USA) has been one of the most commonly used toric IOLs. Several previous studies presented its perfect astigmatism correction efficacy [4–7], but this efficacy can be affected by many factors. Surgical incision and surgically induced astigmatism (SIA) [8] may be one of the most important factors [9]. Currently, there is no agreement concerning the incision for toric IOL implantation. Theoretically, clear corneal incision through steep meridian could decrease some of the corneal astigmatism [10] without changing the astigmatic axis and thus will not induce irregular astigmatism to effect visual quality after the surgery, which has been chosen by more and more surgeons. However, this has not been verified by clinical studies, and there are still many surgeons making non-steep-axis clear corneal incisions basing on their habits such as temporal, superior incisions et al. [5, 11].

Therefore, the purpose of this prospective study is to compare the clinical outcomes in terms of visual acuity, residual astigmatism, accuracy of preoperative calculation of toric IOL and visual quality in eyes undergoing cataract surgery with AcrySof Toric IOLs implantation using these two types of incisions. Our results should provide guidance of future cataract surgery with toric IOLs implantation.

## Methods

This study was approved by the Institutional Review Board of the Eye and ENT Hospital of Fudan University, Shanghai, China, and was registered at www.clinicaltrials.gov (accession number NCT02182921). All procedures adhered to the tenets of the Declaration of Helsinki. Each patient involved in this study has written an informed consent.

Sixty patients who underwent uneventful phacoemulsification combined with AcrySof Toric IOL (Alcon Laboratories, Inc., Fort Worth, TX, USA) implantation between September 2014 and April 2015 in Eye and ENT Hospital of Fudan University were enrolled. Inclusions were cataract with regular corneal astigmatism more than 1.0D. Exclusion criteria were ophthalmic pathology that might affect postoperative visual function, such as corneal diseases, glaucoma, ocular trauma et al. Patients with intraoperative or postoperative complications were also ruled out. The patients were divided into two groups (30 eyes of 30 patients in each group): steep-axis incision group, and non-steep-axis incision group.

### Preoperative examinations

Routine preoperative examinations were performed on every patient enrolled in this study, including uncorrected visual acuity (UCVA) and best corrected visual acuity (BCVA), slit-lamp examination, corneal topography (Pentacam HR; OCULUS Optikgeräte, Wetzlar, Germany), B-scan ultrasonography, IOL spherical power calculation (SRK/T formula from IOLMaster 5.0; Carl Zeiss AG, Oberkochen, Germany). Online toric calculator software (Alcon, Inc., accessible at http://www.acrysoftoriccalculator.com) was utilized to calculate the IOL cylinder power, taking into account keratometry data (total corneal curvature from Pentacam), SIA and the position of the incision.

### Surgical techniques

Before the surgery, the patient was instructed to sit at the slitlamp with head straight in the chinrest and eyes focusing horizontally ahead. The location of incision and axial position of IOL were marked on the cornea close to the limbus using a sterile marker pen under the illumination of slit beam.

Surgeries were performed by one experienced surgeon (Prof. Yi Lu) under topical anesthesia in a standardized manner. For patients in steep-axis incision group, a clear corneal incision of 2.6 mm was made on the steep axis, while for patients in non-steep-axis incision group, an incision of the same size was made 15–75° deviating from steep axis. Subsequent procedures were all the same between the two groups as follows: injection of viscoelastics into anterior chamber, 5.5 mm continuous curvilinear capsulorhexis, hydro-dissection, phacoemusification, irrigation and aspiration of cortex, implantation of toric IOL, aspiration of viscoelastics, clockwise rotation of IOL towards planned axis, and finally incision hydration. No suture was used. The post-op therapies for all eyes were levofloxacin (Cravit; Santen Pharmaceutical) and prednisolone acetate eye drops (Allergan Pharmaceutical Ireland, Westport, Ireland) four times a day for 2 weeks, and pranoprofen (Pranopulin; Senju Pharmaceutical, Osaka, Japan) four times a day for 1 month.

### Postoperative assessment

Postoperative examinations were conducted 3 months after surgery to assess visual acuity, residual astigmatism, corneal astigmatism, SIA (obtained by online calculator at http://sia-calculator.com) and toric IOL rotation. Toric IOL axis was re-calculated according to actual SIA and its deviation from implanted axis was assessed. Visual function including point spread function (PSF), modulation transfer function (MTF) and high-order aberrations (HOAs) were also investigated. Toric IOL rotation was assessed as the method we used before [12]. Visual function was acquired using KR-1 W Wave-Front Analyzer (Topcon Corp., Tokyo, Japan) when the diameter of pupil was no less than 6 mm.

### Statistical analysis

SPSS version 17.0 was used for statistical analysis. Measurement data were shown in form of mean ± SD; Student’s *t* test was performed to compare the differences between two groups while paired t test was used when comparing preoperative and postoperative date in one group. Chi-square test was performed to analyze categorical data. *P*-value <0.05 was considered for statistical significance.

## Results

### Patients’ characteristics

Table 1 displays the demographic characteristics of patients. No statistically significant differences were found between the two groups except position of incision (angular distance between the incision center and steep axis) (all *P* > 0.05, Student’s *t* test or Chi-square test) Table 2 shows the proportion of toric IOL models used in the two groups and no significant differences were found (all *P* > 0.05, Chi-square test).

**Table 1:** Comparisons of patient characteristics between the two groups

Parameter	Steep-axis	Non-steep-axis	*P* value
Age (year)	60.83 ± 12.47	63.90 ± 15.03	0.393
Gender (Male/Female)	10/20	11/19	0.787
Operated eye (Right/Left)	14/16	17/13	0.438
UCVA (logMAR)	0.81 ± 0.31	0.78 ± 0.28	0.684
BCVA (logMAR)	0.55 ± 0.25	0.63 ± 0.25	0.227
AXL (mm)	25.42 ± 2.20	24.70 ± 1.89	0.182
NC grades	2.90 ± 0.53	2.88 ± 0.58	0.908
Corneal astigmatism (D)	2.09 ± 0.55	2.05 ± 0.57	0.790
Incision position (°)^*^	0.00 ± 0.00	29.80 ± 16.33	<0.001

Values are presented as the mean ± standard deviation or *n*

Abbreviations: UCVA = uncorrected visual acuity; BCVA = best-corrected visual acuity; AXL = axial length; logMAR = logarithms of the minimal angle of resolution; IOL = intraocular lens

*Data of incision position was the angular deviation from the steep meridian

No significant differences were found between the two groups except incision position (Student’s *t* test or Chi-square test)

**Table 2:** Proportion of toric IOL models used in the two groups

	T3	T4	T5	T6	T7
Steep-axis	56.67% (17/30)	20.00% (6/30)	13.33% (4/30)	6.67% (2/30)	3.33% (1/30)
Non-steep-axis	43.33% (13/30)	33.33% (10/30)	13.33% (4/30)	10.00% (3/30)	-

Values are presented as percentage (*n*)

### Refractive and corneal statue after surgery

Residual astigmatism of steep-axis and non-steep-axis incision group were −0.61 ± 0.27D and −0.66 ± 0.37D, respectively 3 months after surgery, which was not statistically different (*P* = 0.412, Student’s t test). No difference was either found in toric IOL rotation between the two groups (*P* = 0.980, Student’s *t* test).

In steep-axis incision group, the corneal astigmatism reduced significantly 3 month postoperatively compared with preoperative one (2.09 ± 0.55 vs. 1.59 ± 0.58 D, *P* < 0.001, Paired t test). However, in non-steep-axis incision group, no significant change was found in corneal astigmatism. (2.05 ± 0.57 vs 2.02 ± 0.77 D, *P* = 0.784; Paired *t* test).

Besides, the anterior corneal surfaces of only 10.00% (3/30) of patients in steep-axis incision group were irregular while the proportion was as high as 43.33% (13/30) in non-steep-axis incision group after surgery (*P* = 0.004, Chi-square test).

Angular variation of corneal steep meridian was significantly bigger after surgery in non-steep-axis incision group than in steep-axis incision group (9.85 ± 7.76° and 4.51 ± 2.99°, respectively, *P* = 0.001; Student’s *t* test).

### SIA and deviation between implanted and re-calculated toric IOL axis

At 3 month after surgery, SIA of steep-axis and non-steep-axis incision group were 0.50 ± 0.21D and 0.54 ± 0.25D respectively with no statistical difference (*P* = 0.461; Student’s *t* test). However, when taking actual SIA and incision position into consideration to re-calculate the toric IOL axis, the gap between the original and the theoretical axis was significantly larger in non-steep-axis incision group (0.00 ± 0.00° and 2.79 ± 2.02°, respectively; *P* < 0.001; Student’s *t* test).

### Visual acuity and visual function after surgery

As shown in Table 3, UCVA and BCVA improved statistically in both groups after surgery (all *P* < 0.001, Paired *t* test). No significant differences were found in visual acuity between the two groups after surgery (both *P* > 0.05; Student’s *t* test).

**Table 3:** Preoperative and postoperative visual acuity of the two groups

		Steep-axis	Non-steep-axis	*P* value
UCVA (logMAR)	Pre-op	0.81 ± 0.31	0.78 ± 0.28	0.684
	Post-op	0.27 ± 0.20^*^	0.31 ± 0.22^*^	0.427
BCVA (logMAR)	Pre-op	0.55 ± 0.25	0.63 ± 0.25	0.227
	Post-op	0.08 ± 0.09^*^	0.10 ± 0.08^*^	0.386

Values are presented as the mean ± standard deviation

Abbreviations: UCVA = uncorrected visual acuity; BCVA = best-corrected visual acuity; logMAR = logarithms of the minimal angle of resolution; Pre-op = preoepration; Post-op = postoperation

*Visual acuity improves significantly after surgery in both groups (Paired *t* test, all *P* < 0.05)

**Fig. 1:**
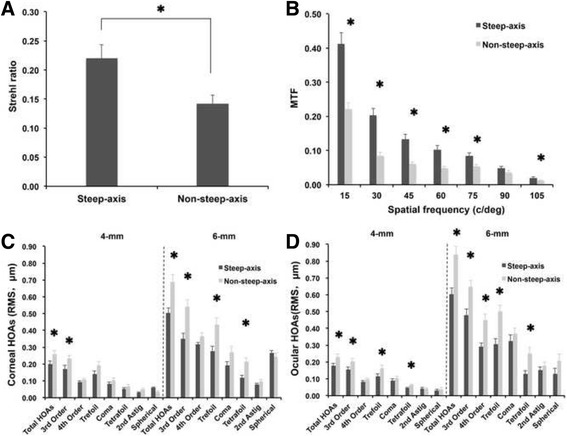
Comparisons of visual quality between the two groups 3 months postoperatively. **a** PSF, (**b**) MTF, (**c**) Corneal HOAs (**d**) Ocular HOAs. * Significant difference was found between the two groups (all *P* < 0.05, Student’s *t* test). Abbreviations: PSF = Point spread function; MTF = Modulation transfer function; HOA = Higher-order aberrations; Astig = Astigmatism; c/deg. = Cycles/degree; RMS = Root mean square

However, performances of visual function were better in steep-axis group as shown in Fig. 1. PSF of each group was 0.22 ± 0.13 and 0.14 ± 0.08 respectively, with significant difference (Fig. 1a; *P* = 0.006; Student’s *t* test). MTFs of patients in the two group were significantly different in all spatial frequency except 90 c/deg. (Fig 1b, 15: *P* < 0.001, 30: *P* < 0.001, 45: *P* < 0.001, 60: *P* < 0.001, 75: *P* = 0.003, 105: *P* = 0.022; Student’s *t* test).

At 3 month after surgery, both corneal and ocular HOAs were greater in non-steep-axis incision group. For corneal HOAs, total HOAs and 3rd order HOAs were significantly different between the two groups at both 4 mm and 6 mm size and the differences in trefoil and tetrafoil aberrations were also significant at 6 mm size (Fig. 1c, all *P* < 0.05, Student’s *t* test). For ocular HOAs, total HOAs, 3rd order HOAs, trefoil and tetrafoil aberrations were significantly different between the two groups at both 4 mm and 6 mm pupil size and the differences in 4th order HOAs were also significant at 6 mm pupil size (Fig. 1d, all *P* < 0.05, Student’s *t* test).

## Discussion

Toric IOL has been proved to have perfect astigmatism correction effect. However, its efficacy is being affected by many factors, among which incision location and SIA are of great importance. Relatively, steep-axis corneal incision might be preferable due to its decrease of corneal astigmatism without changing the astigmatism axis. Nevertheless, owing to personal surgical practice, some surgeons tend to choose a habitual incision location. Studies comparing different incision locations’ influences on astigmatism correction after toric IOL implantation were also scant, let alone studies concentrating on postoperative visual quality. Therefore, we comprehensively assessed the clinical outcomes after implantation of AcrySof Toric IOLs with steep-axis incision and non-steep-axis incision. And we found that compared to patients with non-steep-axis incision, significantly smaller postoperative corneal astigmatism, lower postoperative irregular astigmatism, and better visual quality were found in patients with steep-axis incision.

Incision’s influences on clinical outcomes of toric IOL implantation majorly depend on its influences on corneal astigmatism. Incisions of different sizes, directions, and types will lead to SIA of various vector magnitudes [13–16]. Although the incision sizes adopted these days are small and even micro [5, 17, 18], its interference to cornea is not insignificant. Utilization of appropriate incision location might help to reduce corneal astigmatism intraoperatively [10, 19], and amounts of astigmatism could be largely reduced in combination with toric IOL implantation.

Previous studies hypothesized that incision at the steep axis could flatten the steep meridian and steepen the flat meridian [10], and theoretically correct partial astigmatism without changing axial direction. In our study, corneal astigmatism in steep-axis incision group decreased significantly as much as 0.40 ± 0.52D at 3 months after surgery, which confirmed the relaxation of cornea by steep-axis incision in the steep meridian. In addition, we also found that steep-axis incision exerted subtler impact on the axis of corneal astigmatism compared with non-steep-axis incision group: 5.78 ± 5.12° vs 10.13 ± 8.35° (statistically smaller) 3 months postoperatively. Consequently, steep-axis incision could reduce the cylinder power of toric IOL. According to previous studies, the residual astigmatism caused by toric IOL rotation was positively correlated with its cylinder power [20]. Thus, lower cylinder power of toric IOL was likely to have better results, which indirectly proves that steep-axis incision is better for toric IOL implantation.

Non-steep-axis incision was found to give rise to irregular astigmatism in our study, which corresponded with the results of visual quality. Since incisions nowadays are small or even micro in cataract surgery, the value of SIA is not large enough to affect visual acuity or refractory status obviously but may affect visual quality, and the increase of irregularity of corneal shape may also worsen the corneal aberration and consequently the overall visual quality. Previous studies showed that wavefront analysis might predict visual complaints. For example, glare was found to be associated with total HOAs and spherical aberration [21–23]. In our study, we found better HOAs results together wth higher PSF and MTF data in patients with steep-axis incision, which indicating a better visual quality.

In conclusion, steep-axis incision could reduce the cylinder power of toric IOL and induce less irregular astigmatism, which brings about better postoperative visual quality. Therefore, steep-axis incision may be an ideal incision choice for toric IOL implantation.

## Abbreviations

AXL: Axial length
BCVA: Best corrected visual acuity
HOAs: High-order aberrations
IOL: Intraocular lens
logMAR: logarithms of the minimal angle of resolution
MTF: Modulation transfer function
PSF: Spread function
RMS: Root mean square
SIA: Surgically induced astigmatism
UCVA: Uncorrected visual acuity
